# Knowledge structure and emerging trends of CAR-T therapy in breast cancer: A bibliometric analysis of the Web of Science Core Collection (2012–2025)

**DOI:** 10.1097/MD.0000000000051342

**Published:** 2026-10-30

**Authors:** Xue Liu, Yan Gao, Zhiqiang Qu, Yaxin Zhou, Ruiping Jia, Huan Liu, Changying Wang, Huabao Xiong

**Affiliations:** a School of Basic Medical Sciences, Jining Medical University, Jining, Shandong, China; b Library, Jining Medical University, Jining, Shandong, China; c Department of Practical Teaching Management, Jining Medical University, Jining, Shandong, China; d College of Clinical Medicine, Jining Medical University, Jining, Shandong, China; e Institute of Immunology and Molecular Medicine, Jining Medical University, Jining, Shandong, China; f Jining Key Laboratory of Immunology, Jining Medical University, Jining, Shandong, China.

**Keywords:** bibliometric analysis, breast cancer, CAR-T, CiteSpace, immunotherapy

## Abstract

**Background:**

Chimeric antigen receptor T-cell (CAR-T) has shown clinical efficacy in hematological malignancies and is being explored for application in breast cancer. However, although the literature in this field is growing rapidly, there is still a lack of systematic bibliometric review of its disease-specific knowledge structure, research hotspots and development trends.

**Methods:**

We retrieved literature on CAR-T therapy for breast cancer published between 2012 and 2025 in the Web of Science Core Collection. Bibliometric tools such as CiteSpace were used to perform bibliometric and visual analyses across multiple dimensions, including publication volume, journals, authors, institutions and countries, keywords, subject categories, and co-citations of references.

**Results:**

The annual number of related publications shows a sustained increasing trend. Harvard University and its affiliated hospitals rank first globally in terms of both the number of published papers and their centrality. “chimeric antigen receptors” and “adoptive immunotherapy” hold a core position. The cutting-edge topics cover the progress and targeted treatments of triple-negative breast cancer. The reference co-citation analysis yielded 53 clusters, covering topics such as solid tumors, antitumor efficacy and tumor microenvironment, reflecting the knowledge base in this field.

**Conclusions:**

This study depicts the research pattern and evolution trend of CAR-T therapy in the field of breast cancer. The research focus has shifted from early feasibility exploration to directions such as tumor microenvironment, heterogeneity of target antigens, and combination therapy. Future research can focus on cross-institutional collaboration, expansion of target points, and optimization of CAR structure.

## 1. Introduction

Breast cancer is the cancer with the highest incidence and mortality rates among women worldwide. According to GLOBOCAN 2024 estimates, approximately 2.4 million new cases and 694,000 deaths occurred globally, accounting for 24.3% of all new cancer cases and 16.2% of all cancer deaths in women.^[1]^ Despite the continuous progress of traditional methods such as surgery, chemotherapy, endocrine therapy, and molecular targeted therapy, treatment resistance, metastasis, and recurrence remain the main causes of patient death. Therefore, it is urgent to explore new treatment strategies to improve therapeutic effects and effectively inhibit tumor recurrence and metastasis.

Against this backdrop, chimeric antigen receptor T-cell (CAR-T) therapy, as a revolutionary immunotherapy strategy, has achieved promising success in hematological malignancies, bringing new hope for the treatment of solid tumors including breast cancer.^[2–4]^ The complete remission rate for CAR-T therapy in B-cell acute lymphoblastic leukemia (ALL) can reach 90%.^[5,6]^ However, when CAR-T therapy is applied to solid tumors such as breast cancer, it faces multiple challenges such as a complex tumor microenvironment (TME), high antigen heterogeneity, T-cell homing, and on-target/off-tumor toxicity, and its therapeutic effect is far inferior to that in hematological malignancies.^[7–11]^

Bibliometrics achieves scientific evaluation by systematically analyzing the content and structural features of academic publications.^[12,13]^ In recent years, this method has been widely applied to analyze the research landscape in tumor immunotherapy. For instance, Li et al, based on large-scale data mining, systematically depicted the knowledge map of T cell exhaustion research, identified research hotspots such as immune checkpoints, transcriptional regulation, metabolic reprogramming, and single-cell omics, and pointed out that epigenetic editing, metabolic intervention, and genetic engineering modification are emerging frontiers.^[14]^ However, this study focused on the immune functional state of T cell exhaustion itself and did not specifically explore the disease-specific transformation challenges of CAR-T therapy in breast cancer, which is a highly heterogeneous solid tumor. Furthermore, at the overall level of the CAR-T field, Sweileh et al conducted a bibliometric analysis of the CAR-T cell products approved by the FDA.^[15]^ The study found that the number of published papers related to CAR-T therapy for hematological malignancies increased by an average of 63.4% each year, with a total citation count exceeding 57,000 and an H-index reaching 103. There was extensive international cooperation, with European countries being particularly highly dependent on international collaboration. The research topics gradually expanded from CD19-targeted products to B-cell maturation antigen-targeted products, and new directions such as cost-benefit analysis and real-world validation emerged. These indicators and the evolution of research themes together reflect the fundamental discoveries and sustained research interests in the CAR-T field.

Based on this, this study differs from previous bibliometric studies that conducted extensive analyses on various solid tumors. The study uses the Web of Science Core Collection (WoSCC) as the data source and employs bibliometric tools such as CiteSpace to conduct co-occurrence and clustering analysis of countries, institutions, authors, and keywords. The aim is to reveal the current research status, knowledge structure, key hotspots, and development trends in this field, providing empirical references for strategic planning, key direction selection, and collaboration network construction of future research, as well as data support for the clinical translation research of breast cancer CAR-T therapy.

## 2. Methods

### 2.1. Data sources

We focused on the research literature of CAR-T therapy in the treatment of breast cancer. The data source was the WoSCC database. Literature was retrieved using the search string “CAR-T” AND “Breast cancer.” To focus on the original research results, only “Article” and “Review” were included. The search period was up to July 29, 2025, with no time range restrictions set. Duplicate literature, irrelevant publications, editorials, and case reports were excluded to ensure dataset relevance and validity. Finally, 508 publications were identified for inclusion.

### 2.2. Research method

We utilized the CiteSpace (version 6.3.R1) knowledge graph analysis tool to conduct a multi-time series dynamic visualization study on the retrieval results of the WoSCC database. Through multiple dimensions, such as the temporal and spatial distribution characteristics of the literature, keyword analysis, topic category analysis, and co-citation analysis of references, the research trajectory and development trend of CAR-T therapy in the field of breast cancer were systematically explored.

The specific operation process is as follows (Fig. 1). Data retrieval and screening were independently conducted by 2 researchers using the WoSCC database. All records, including complete records and references, were exported in plain text format. The search results were then imported into CiteSpace 6.3.R1 software for analysis, strictly following standard processes: Time Slicing, Pruning, Modeling, Thresholding, Merging, and Mapping. In detail, time zone segmentation (Time Slicing) was set from 2012 to 2025 with a 1-year interval to capture annual variations in research activity while preserving network clarity. Node screening was carried out using the modified G-index (*k* = 25). This setting achieves an optimal balance between core node identification and network topological readability, taking into account both the revelation of key knowledge structures and the degree of visual analysis. The node types were set in sequence as Author, Institution, Country, and Keyword. The “Pathfinder” algorithm and the “Pruning the merged network” option were selected to optimize the network structure and highlight the key features. The remaining parameters remained at the default configuration. This parameter combination could effectively balance the complexity of the network and the visualization effect, ensuring the objectivity and reliability of the analysis results. Centrality refers to the intermediate centrality calculated by CiteSpace, which measures the extent to which a node connects separate parts of the network; the higher the value, the stronger the bridging role, but it should not be interpreted as direct evidence of method quality or clinical efficacy. Larger nodes indicate a higher frequency. The thickness of the edges between nodes represents the strength of cooperation, co-occurrence, or co-citation relationships, depending on the specific network.

**Figure 1.**
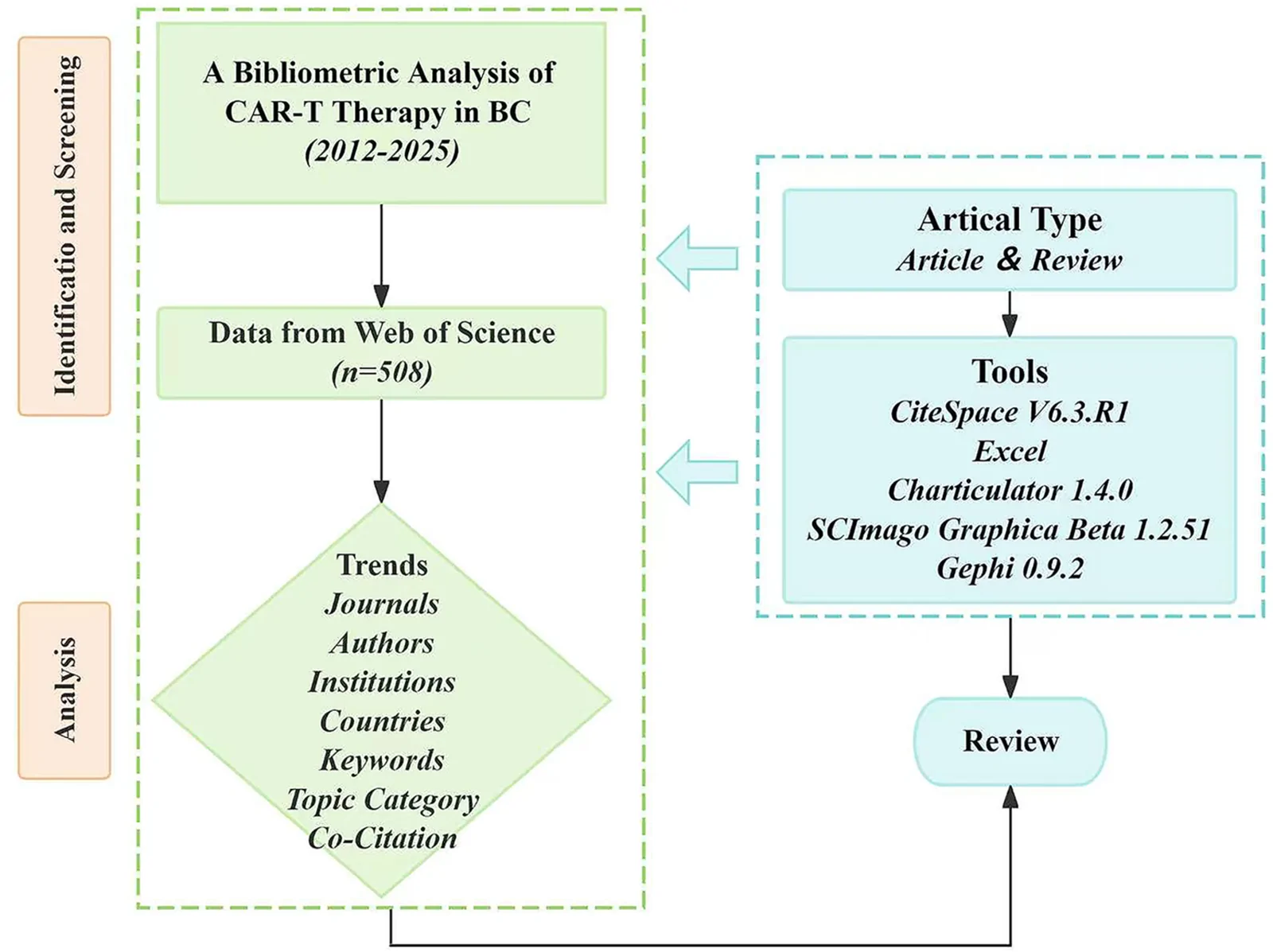
Flowchart of this study. CAR-T = chimeric antigen receptor T-cell.

## 3. Results

### 3.1. Temporal distribution characteristics of the literature

The temporal distribution characteristics of academic literature can effectively reflect the development trend of a research field. The annual distribution pattern of the number of published papers related to CAR-T therapy in the treatment of breast cancer is shown in Figure 2A. There have been research reports in English literature since 2012, and the overall growth trend has been continuous. As of July 29, 2025, the number of published articles for this year has reached 61. A polynomial fitting was conducted on the cumulative number of publications (the total number of publications from 2012 to 2025), and the obtained *R*^2^ value was 0.9301. The blue dotted line in the figure represents the fitting curve, further confirming the upward trend of the number of publications. This indicates that research on CAR-T therapy in the treatment of breast cancer has received extensive attention and emphasis from the international academic community. Especially after 2020, the growth rate of the number of publications has accelerated. We attribute the post-2020 publication surge to 3 factors: the success of mRNA technology during the pandemic bolstered confidence in gene-modified cell therapies; the clinical validation of CD19-targeted CAR-T products generated momentum that subsequently spilled over into solid tumors; and the increasing policy support for CAR-T therapy in the field of breast cancer.

**Figure 2.**
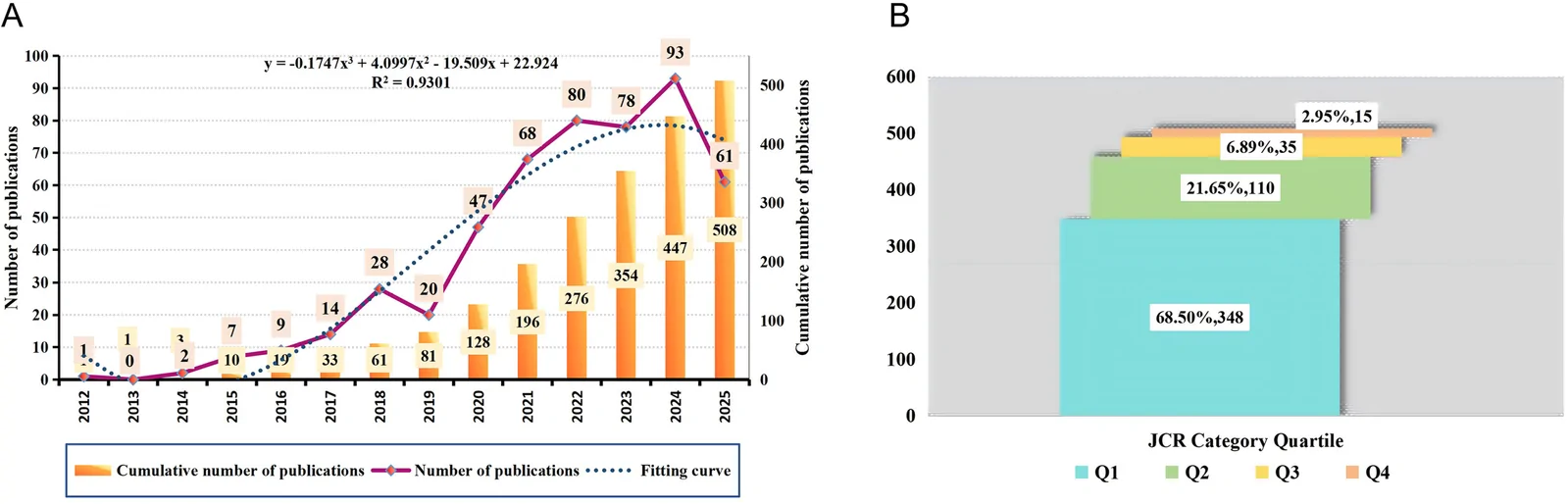
(A) Trends in the number of publications from 2012 to 2025. (B) Distribution of publications by JCR category quartile. JCR = Journal citation reports.

### 3.2. Spatial distribution characteristics of the literature

#### 3.2.1. Analysis of journals and co-cited journals

The final dataset comprised 508 publications, with 233 articles and 275 reviews. These 508 documents were published in 223 journals. The journals are divided into quartiles (Q1–Q4) within each subject category (Fig. 2B). Q1 represents journals with the top 25% performance ranking, Q2 represents the next 25%, and Q3 and Q4 represent the decreasing tiers of rankings. The number and proportion of papers in Journal citation reports divisions are shown in Figure 2B. There are 348 papers published in Q1, accounting for 68.50%; 110 papers in Q2 (21.65%); 35 papers in Q3 (6.89%); and 15 papers in Q4 (2.95%). Among the 508 publications in the Journal citation reports divisions, there is a significant gradient distribution. Q1 and Q2 together account for 90.15% of the total, with a lower proportion in the lower sections, indicating that the overall academic quality of the included papers is high, and the mainstream research results are concentrated in the top influence ranks.

Analysis of journals and co-cited academic journals. Table 1 lists the top 10 journals with the highest number of citations, ranked by h-index. These top 10 journals have published a total of 176 publications, accounting for 34.65% of all the included publications. The journals “Cancers” and “Frontiers in Immunology” rank at the top. The journal “Journal of Hematology & Oncology” has the highest impact factor, at 47.8, highlighting its significant relevance in this field. It is notable that most of these journals belong to the Q1 and Q2 categories. The journal “Frontiers in Immunology” also has the largest number of NP (38), ranking first in the total number of citations (2170).

**Table 1:** Bibliometric indicators of high-impact journals.

Source	h_index	IF 2025	JCR 2025	TC	NP	PY_start
*Cancers*	21	4.8	Q2	1225	31	2020
*Frontiers in Immunology*	21	7.0	Q1	2170	38	2017
*International Journal of Molecular Sciences*	16	5.6	Q1	736	25	2017
*Journal for Immunotherapy of Cancer*	15	11.7	Q1	955	21	2018
*Journal of Hematology & Oncology*	11	47.8	Q1	1942	11	2016
*Molecular therapy*	10	11.4	Q1	574	12	2014
*Cancer Immunology Immunotherapy*	9	5.8	Q1	357	9	2018
*Frontiers in Oncology*	9	3.4	Q2	601	13	2019
*Oncoimmunology*	8	6.2	Q1	540	9	2015
*Biochimica et Biophysica Acta-reviews on Cancer*	6	11.2	Q1	475	7	2021

JCR = Journal citation reports, NP = number of publications, PY_start = PY_start indicates the year the journal started publication, TC = total citations.

Table 2 presents the top 5 journals with the highest co-citation frequency in this field. The journal “Clinical Cancer Research” has the highest co-citation rate (n = 427), while the journal “Advanced Drug Delivery Reviews” has the highest centrality value (0.07). This indicates that the former is a frequently cited knowledge source, while the latter plays a relatively important bridging role in the co-citation journal network. Overall, these findings suggest that authoritative journals related to oncology, immunology, pharmacology, and basic biomedical research collectively constitute important publications and citation sources in this field.

**Table 2:** Cited journals and centrality in breast cancer CAR-T therapy.

Rank	Frequency	Journal	H-Index	IF (2025)	Country	Rank	Centrality	Journal	H-Index	IF (2025)	Country
1	427	*Clinical Cancer Research*	21	10.9	USA	1	0.07	*Advanced Drug Delivery Reviews*	18	21.0	Netherlands
2	422	*Cancer Research*	20	22.6	USA	2	0.06	*Anticancer Research*	7	1.8	Greece
3	352	*New England Journal of Medicine*	83	84.5	USA	3	0.06	*Immunological Reviews*	16	10.6	England
4	349	*Proceedings of the National Academy of Sciences of the United States of America*	38	9.5	USA	4	0.05	*British Journal of Cancer*	16	7.8	England
5	348	*Frontiers in Immunology*	39	7.0	Switzerland	5	0.05	*Cancer Letters*	25	11.8	Netherlands

CAR-T = chimeric antigen receptor T-cell.

A dual-map overlay graph was created using CiteSpace to visually display the relationship between citing journals and cited journals (Fig. 3). The left side represents citing journals, and the right side represents cited journals. The green paths indicate that articles published in “Medicine, Medical, Clinical” journals frequently cite articles published in “Molecular, Biology, Genetics” journals. The orange paths indicate that “Molecular, Biology, Genetics” is frequently cited in “Molecular, Biology, Immunology.”

**Figure 3.**
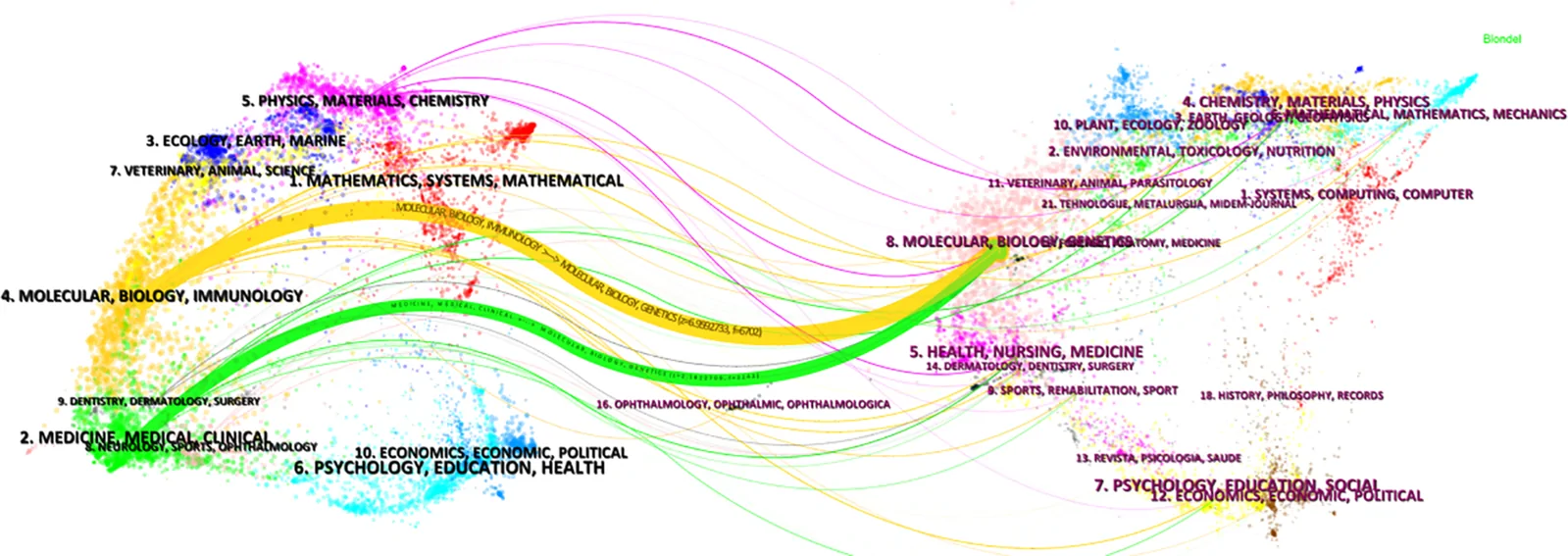
A dual-map overlay of journals.

#### 3.2.2. Bibliometric analysis of author distribution

In our analysis of the authors’ cooperative networks (Fig. 4), the node size represents the number of published papers, the thickness of the connection lines reflects the intensity of cooperation, and the color gradation of the connection lines indicates the dimension of cooperation time. Collaborative research among productive scholars is conducive to deepening the theoretical system and practical application of CAR-T therapy for breast cancer. Several core research clusters have been formed at present. The network parameters show that the number of nodes is 334, the number of connections is 500, and the network density is 0.009 (lower than the conventional threshold of 0.1). The *Q* value is 0.9231. A *Q* value >0.3 indicates that the obtained network community structure is meaningful, and the closer its value approaches 1, the better the clustering of the network is achieved. In Figure 4, there are few and scattered connections among the core authors, and the node density is low. To facilitate the observation of the network diagram, we set the threshold at 2, meaning that only authors who have published more than 2 articles are shown in the diagram. The graph indicates that, apart from the relatively large collaboration network formed by the high-productivity authors at the center of the network, other authors publish their articles either individually or in small groups.

**Figure 4.**
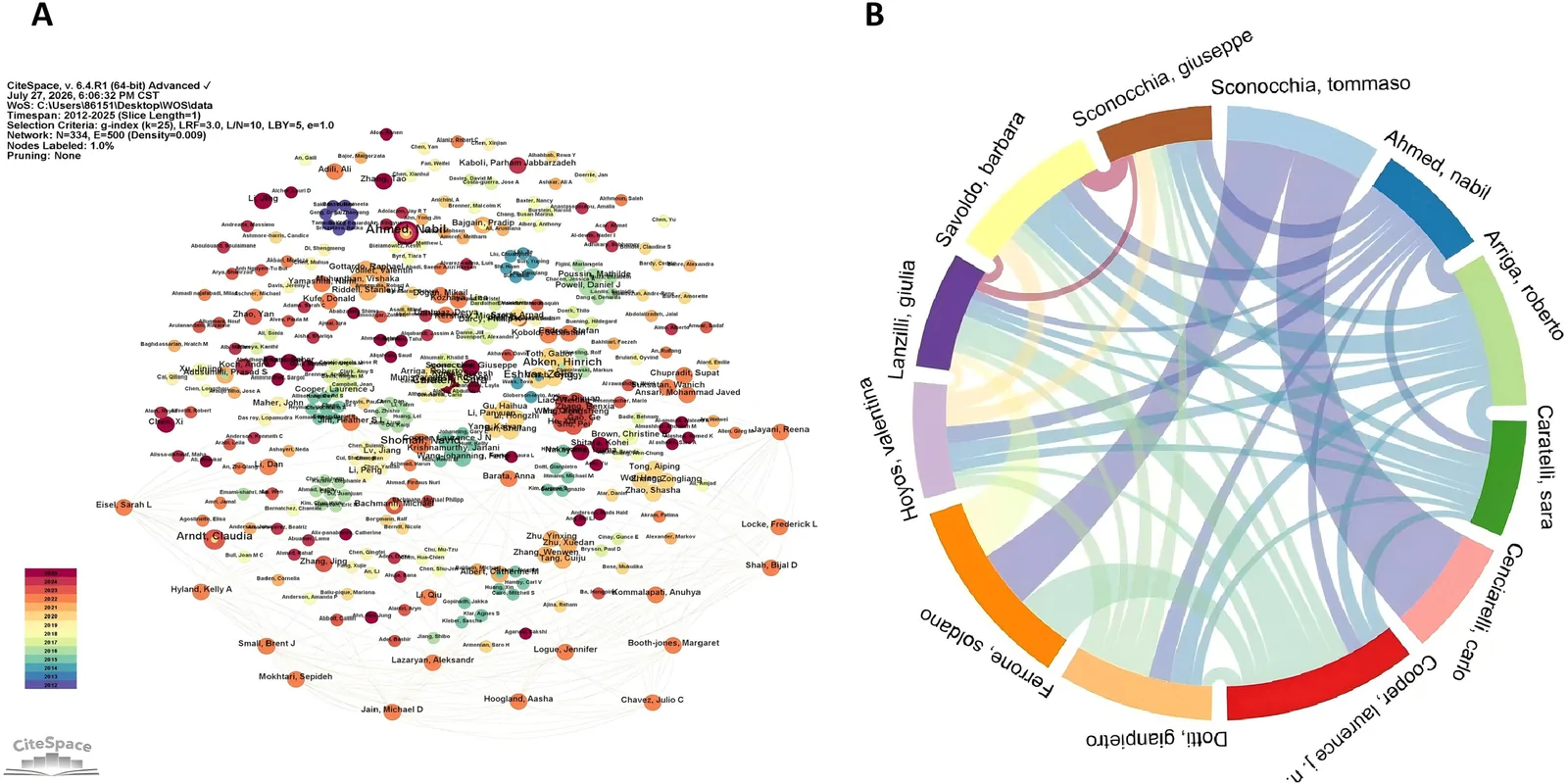
Co-occurrence knowledge graph of literature authors. (A) Co-authorship network by CiteSpace. (B) Charticulator chord diagram.

#### 3.2.3. Bibliometric analysis of institutional and country distribution

Institutions with a publication volume of 10 or more articles in the English-language database are shown in Table 3 and Figure 5. Harvard University and its affiliates emerged as the most active institution in the academic database. It demonstrated outstanding performance in both publication output (a total of 28 articles) and centrality (0.23 and 0.18, respectively), indicating extensive academic influence. The concentrated output in 2017, in particular, may be related to its researchers’ disciplinary advantages or international cooperation. Shanghai Jiao Tong University (13 articles) and Fudan University (10 articles) also ranked among the top, but their centrality was generally low (0.05 and 0.19, respectively), indicating that there is still room for improvement in their international influence. Overall, institutions in developed countries, represented by the US, demonstrated a stronger mediating role in this network. Meanwhile, Chinese institutions, while maintaining a high level of output activity, still have the potential to expand the depth of international cooperation.

**Table 3:** Institutions with output ≥ 10 in the English database.

Rank	Publications	Centrality	Year	Institution
1	15	0.23	2017	Harvard University
2	13	0.18	2017	Harvard University Medical Affiliates
3	13	0.05	2019	Shanghai Jiao Tong University
4	12	0	2015	University of Texas System
5	11	0.11	2015	State University System of Florida
6	11	0	2016	Sichuan University
7	10	0.19	2016	Fudan University
8	10	0.09	2015	University of London
9	10	0.02	2017	Harvard Medical School
10	10	0	2017	Huazhong University of Science & Technology

**Figure 5.**
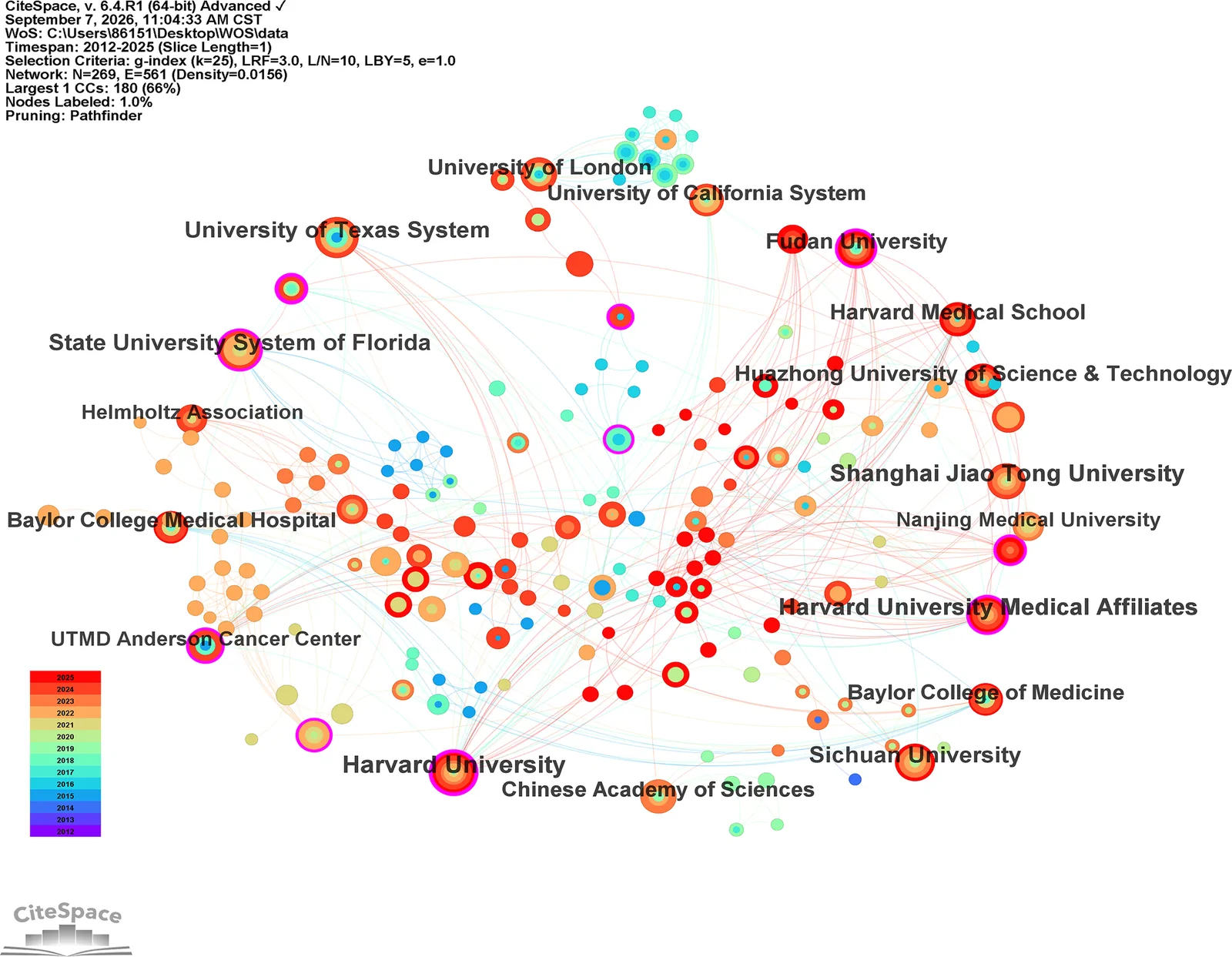
Institutional collaboration map of literature on the relationship between breast cancer and CAR-T therapy. CAR-T = chimeric antigen receptor T-cell.

From the perspective of the national/regional distribution characteristics of global breast cancer CAR-T therapy research (Table 4 and Fig. 6), the United States (179 articles) and China (171 articles) lead in the total number of published articles. In contrast, the United States has higher centrality (0.2) and extensive international cooperation (Degree = 5). The degree of cooperation refers to the number of direct cooperative relationships established by a country with other countries within the cooperative network. It reflects the breadth of the country’s international scientific research cooperation. Germany (with 46 articles) exhibits a significantly higher centrality (0.6) than its publication volume would suggest, indicating that it plays a crucial “bridge” role in the cross-border cooperation network. It is worth noting that Iran (28 articles) and France (15 articles) have enhanced their academic influence through a high degree of international cooperation (Degree = 5 and 6, respectively).

**Table 4:** Countries with output ≥ 10 in the English database.

Rank	Publications	Degree	Centrality	Country	Year
1	179	5	0.2	USA	2012
2	171	1	0	China	2014
3	46	5	0.6	Germany	2016
4	29	2	0.03	Italy	2016
5	28	5	0.25	Iran	2017
6	28	5	0.17	England	2015
7	27	1	0	India	2019
8	15	6	0.22	France	2017
9	15	2	0.06	Canada	2017
10	12	3	0.13	Switzerland	2015
11	12	2	0.06	Poland	2020
12	11	9	0.48	Saudi Arabia	2017
13	11	2	0.06	Spain	2020
14	10	1	0	Japan	2012

**Figure 6.**
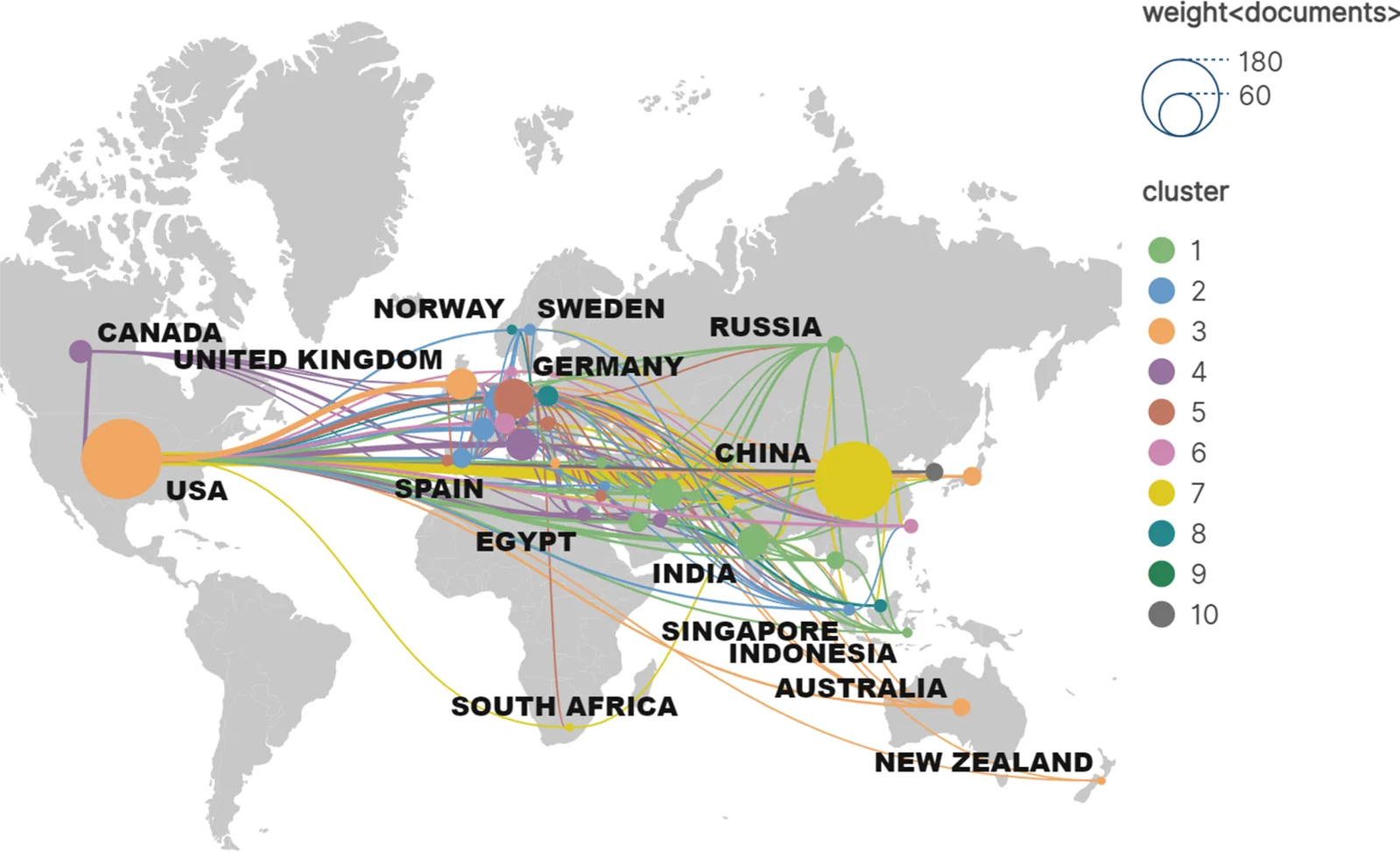
Country/region collaboration map of literature on CAR-T therapy for breast cancer. CAR-T = chimeric antigen receptor T-cell.

### 3.3. Analysis of keywords

#### 3.3.1. Analysis of co-occurrence graph of keywords

Keywords are a concise summary of the research topic, and their frequency distribution can effectively reveal the hotspots of the discipline, research trends, and thematic correlations. The merging of keywords is based on semantic similarity and manual judgment, and also refers to the original citation information in the database. For ambiguous entries, 2 researchers independently review and reach a consensus. The co-occurrence analysis of keywords in this study (Fig. 7A) resulted in 341 nodes and 1556 connections, with a network density of 0.0268. After modular testing, the *Q* value reached 0.9231 (> 0.3), indicating a significant clustering structure. The scale of nodes in the graph is positively correlated with the frequency of keywords, visually presenting the distribution characteristics of core research topics within the field.

**Figure 7.**
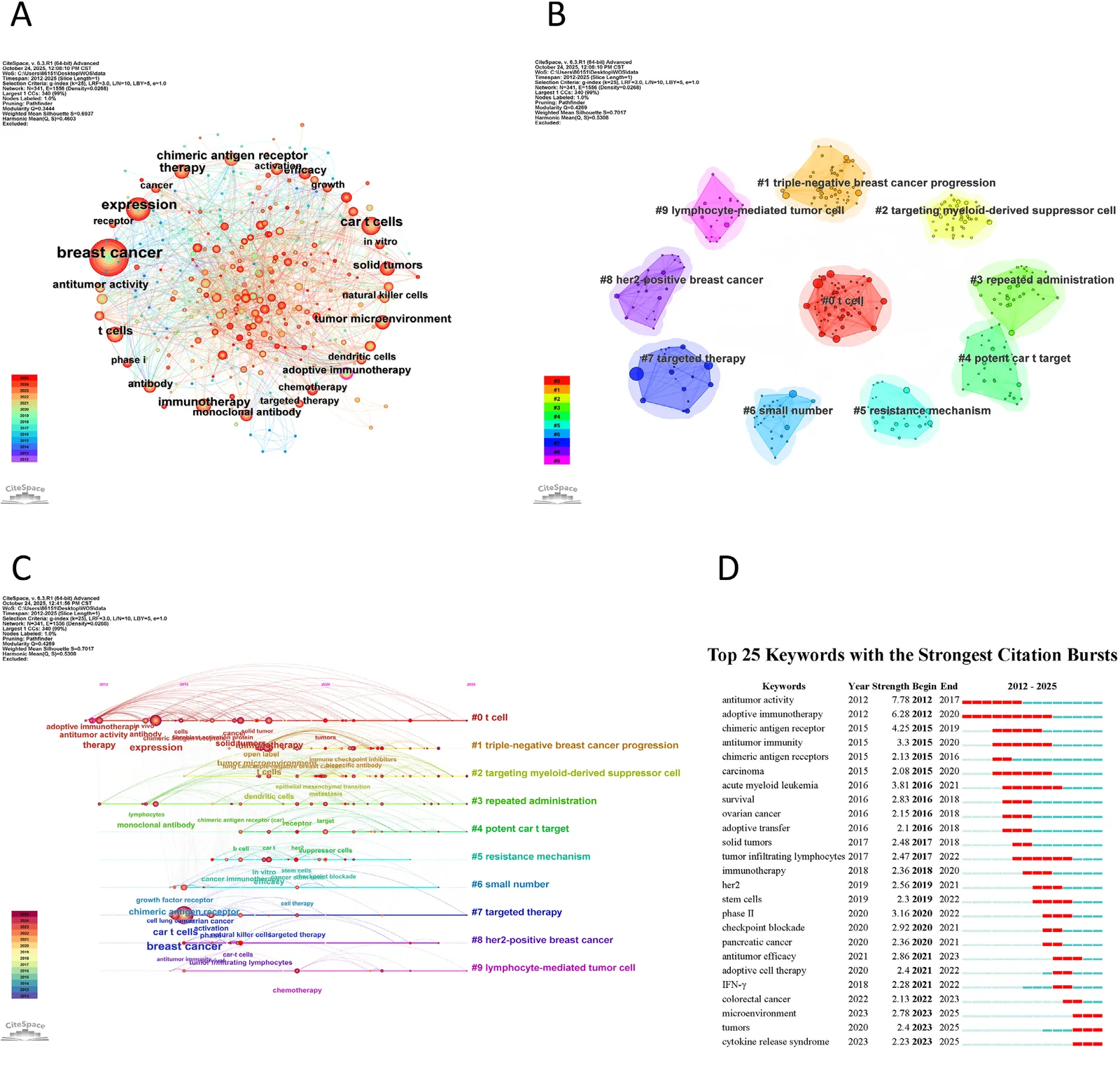
Keyword co-occurrence and clustering analysis. (A) Keyword co-occurrence network; node size and link frequency represent the frequency and co-occurrence intensity of keywords, respectively. (B) Keyword clustering knowledge graph; keywords with high similarity were grouped into the same cluster. (C) Keyword cluster timeline view; clusters are arranged horizontally, and the position of each node indicates the time of keyword emergence. (D) Top keywords with the strongest burst intensity; red segments indicate the burst duration of each keyword.

Table 5 presents the co-occurrence characteristics of keywords in CAR-T therapy research for breast cancer. “breast cancer” (260 times) has the highest frequency of appearance as a basic concept, but its mediating centrality is low (0.02), indicating its universality as a basic research topic. It is notable that “adoptive immunotherapy” (48 times) and “chimeric antigen receptor” (70 times) demonstrated a relatively high centrality (0.13 and 0.1 respectively), playing an important bridging role in the knowledge network.

**Table 5:** Keywords in the English database (frequency ≥30).

Frequency	Degree	Centrality	Keywords	Frequency	Degree	Centrality	Keywords
260	18	0.02	breast cancer	43	38	0.08	antitumor activity
124	36	0.09	expression	43	20	0.02	efficacy
94	22	0.03	car t cells	39	15	0.03	monoclonal antibody
71	22	0.05	therapy	36	48	0.13	adoptive immunotherapy
70	36	0.1	chimeric antigen receptor	35	40	0.09	antibody
68	11	0.02	immunotherapy	33	19	0.02	phase I
56	15	0.03	t cells	31	27	0.06	dendritic cells
49	16	0.01	solid tumors	31	26	0.05	growth
48	19	0.03	tumor microenvironment	30	19	0.03	activation

The keyword co-occurrence network presents a hierarchical structure from basic research to clinical translation, which can be roughly divided into the basic research layer (such as “expression”), the technical method layer (such as “monoclonal antibody”) and the clinical translation layer (such as “Phase I”). Among them, the keyword “tumor microenvironment” has a high occurrence frequency (48 times), with an intermediate centrality of 0.03, suggesting that the research focus is gradually extending from the expression of a single molecule to complex regulatory factors such as the TME. This keyword may have the potential to become an emerging research hotspot.

#### 3.3.2. Analysis of keyword clustering graphs

The CiteSpace software was used to conduct cluster analysis on the keywords, and the log-likelihood ratio algorithm was employed to extract the cluster labels.

Through the CiteSpace keyword clustering analysis, a total of 10 keyword clusters were obtained (Fig. 7B). The modularity *Q* = 0.4269 (> 0.3) and the average silhouette coefficient *S* = 0.7017 (> 0.7) indicate a significant clustering structure with high homogeneity within the clusters. The results are reliable and have explanatory significance. Different colors in the graph represent different clusters, and the cluster labels reveal cutting-edge topics such as “the progression of triple-negative breast cancer” and “targeted therapy.” In hematological malignancies, targeted therapy and CAR-T have a synergistic integration and sequential complementarity relationship.^[16,17]^ In breast cancer, targeted therapy remains the mainstream.^[18–20]^ The combination of CAR-T and targeted therapy is still in the exploratory stage, and the clinical relationship awaits further verification.^[21]^ The size and color depth of the nodes reflect the frequency of the keywords and their temporal evolution, and the connections between the nodes represent the co-occurrence relationship of the keywords. Among them, cluster #0 contains the most keywords, and the overlapping nodes reflect the co-occurrence characteristics across clusters. The knowledge graph visually presents the research orientation centered on T cells. This discovery provides a directional guidance centered on the regulation of T cell function for the clinical transformation of CAR-T therapy in breast cancer.

#### 3.3.3. Keyword timezone and burst detection analysis

Keyword timezone and burst detection analysis can effectively reveal the historical evolution characteristics of a research field. The top 25 keywords in terms of emergent intensity were extracted for quantitative analysis, and a keyword clustering timezone map (Fig. 7C) and keyword burst detection map (Fig. 7D) were drawn. In these maps, the intensity and temporal distribution characteristics of the highlighted keywords directly reflect the dynamic evolution process of the research hotspots in this field.

The time zone chart shows that “antitumor activity” and “adoptive immunotherapy” first emerged in 2012,^[22]^ while keywords such as “cytokine release syndrome” did not appear widely until 2023.^[23,24]^ The study published in Clin Cancer Res in 2012 was an early representative work of anti-FITC CAR-T cell technology. It was the first to target HER2-positive breast cancer with CAR-T cells using FITC-labeled antibodies (such as trastuzumab), and verified the antitumor activity and immunotherapy potential in animal models. In bibliometrics, it can be regarded as a milestone starting point for the keywords “anti-tumor activity” and “immunotherapy” in the field of breast cancer CAR-T. The burst detection further reveals that the emergence of “immunotherapy” continued until 2018,^[25–27]^ indicating its long-term attention; “antitumor immunity” also shows a sustained research interest. The peak period of keyword emergence was from 2015 to 2016,^[28–32]^ with a total of 8 emergent words, among which “chimeric antigen receptor” and “antitumor immunity” emerged simultaneously, possibly reflecting the formation of the research paradigm of CAR-T therapy at this stage. From the perspective of emergent intensity, “antitumor activity” has the highest emergent intensity (7.78), and “chimeric antigen receptor” also shows a relatively high intensity, together forming a stable hot group in this field.^[33]^ It is worth noting that “microenvironment” and “cytokine release syndrome” as new keywords emerging in 2023,^[34]^ their emergence suggests that future research may pay more attention to the interaction between TME and clinical toxicity management.

### 3.4. Topic category analysis

The visualization of the co-occurrence network of topic categories shows that there are at least 4 topics (Fig. 8). The category *Oncology* has the highest frequency (229 times), followed by *Immunology* (120 times), *Biochemistry & Molecular Biology* (76 times), *Medicine, Research & Experimental* (65 times), and *Pharmacology & Pharmacy* (52 times; see Table 6). This indicates that these thematic categories have always been the main focus of extensive research on CAR-T therapy in the field of breast cancer, and are the main contributors in this field. Cell Biology ranks highest in the intermediate centrality aspect (0.3), followed by *Oncology* (0.23), *Medicine, Research & Experimental* (0.19), *Pharmacology & Pharmacy* (0.18), and *Biotechnology & Applied Microbiology* (0.17; Table 6). Their bridging role in interdisciplinary research is highlighted. By adjusting the duration of the outbreak to 1 year, 6 types of outbreak intensity were determined (Table 7), and the appearance of these thematic categories began in 2014. According to the outbreak time, *Nanoscience & Nanotechnology* and *Materials Science, Multidisciplinary* represent the cutting-edge research directions of CAR-T therapy in the field of breast cancer.

**Table 6:** Top 10 subject categories ranked by frequency and betweenness centrality.

Subject categories	Frequency	Subject categories	Centrality
*Oncology*	229	*Cell Biology*	0.3
*Immunology*	120	*Oncology*	0.23
*Biochemistry & Molecular Biology*	76	*Medicine, Research & Experimental*	0.19
*Medicine, Research & Experimental*	65	*Pharmacology & Pharmacy*	0.18
*Pharmacology & Pharmacy*	52	*Biotechnology & Applied Microbiology*	0.17
*Cell Biology*	51	*Nanoscience & Nanotechnology*	0.16
*Chemistry, Multidisciplinary*	37	*Chemistry, Multidisciplinary*	0.15
*Biotechnology & Applied Microbiology*	22	*Biochemical Research Methods*	0.05
*Genetics & Heredity*	21	*Biology*	0.04
*Hematology*	20	*Engineering, Biomedical*	0.03

**Table 7:** Top 6 subject categories with the strongest citation bursts.

Subject categories	Year	Strength	Begin	End	2012–2025
*Biotechnology & Applied Microbiology*	2014	2.83	2014	2017	▂▂▃▃▃▃▂▂▂▂▂▂▂▂
*Genetics & Heredity*	2014	2.17	2014	2017	▂▂▃▃▃▃▂▂▂▂▂▂▂▂
*Multidisciplinary Sciences*	2015	1.74	2015	2019	▂▂▂▃▃▃▃▃▂▂▂▂▂▂
*Hematology*	2016	1.52	2016	2019	▂▂▂▂▃▃▃▃▂▂▂▂▂▂
*Nanoscience & Nanotechnology*	2022	1.6	2023	2025	▂▂▂▂▂▂▂▂▂▂▂▃▃▃
*Materials Science, Multidisciplinary*	2022	1.14	2023	2025	▂▂▂▂▂▂▂▂▂▂▂▃▃▃

**Figure 8.**
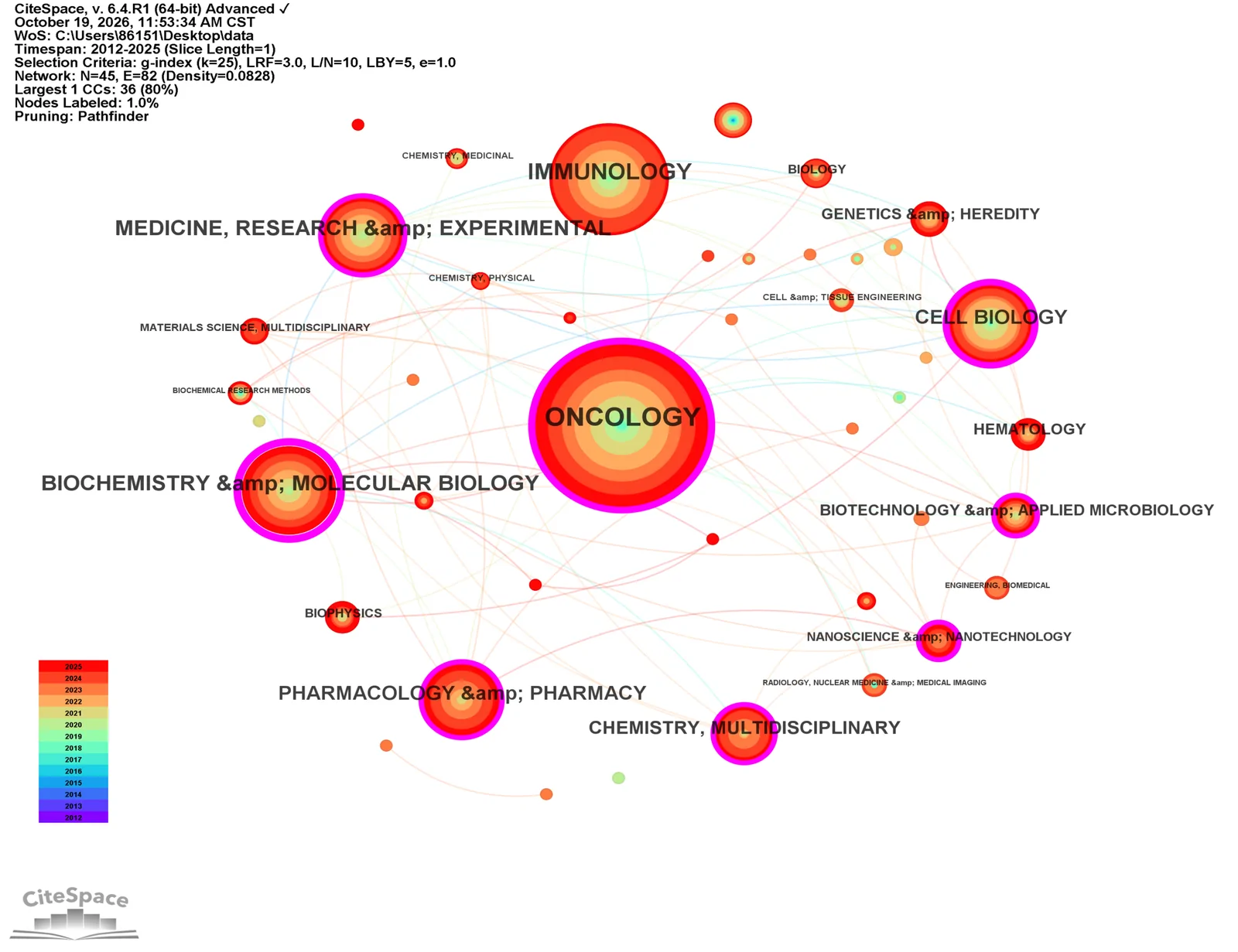
Co-occurrence network map of Web of Science subject categories. Node size and link thickness represent the frequency and intensity of co-occurrence between subject categories, respectively.

### 3.5. Reference co-citation analysis

The co-citation analysis of references reflects the frequently cited papers in the academic research of this discipline and provides basic knowledge support for new research. The fewer the number of clustering labels, the more important the number of papers included in the cluster. Figure 9 shows the co-citation analysis results of 639 references. In the literature clustering analysis, the topic titles of the papers were used as the clustering data source, and the log-likelihood ratio algorithm was employed for clustering analysis. Through the clustering analysis, 53 clusters were obtained (the first 15 clusters are listed in the chart: current status, solid tumor, antitumor efficacy, her2-positive breast cancer, TME, potential therapy, colorectal cancer, driving T cells, novel therapy, various cancer types, therapeutic strategies, progressive mammary cancer, strategies, CAR-T cell checkpoint inhibitor, and killing). The modularity *Q* of the clustering module is 0.7345 (>0.3), and the weighted mean silhouette *S* is 0.8773 (>0.7). The subgroup division is clear. The co-citation clustering analysis indicates that although each cluster seems independent, they are essentially interrelated and collectively form a complex network in the field of breast cancer CAR-T therapy research.

**Figure 9.**
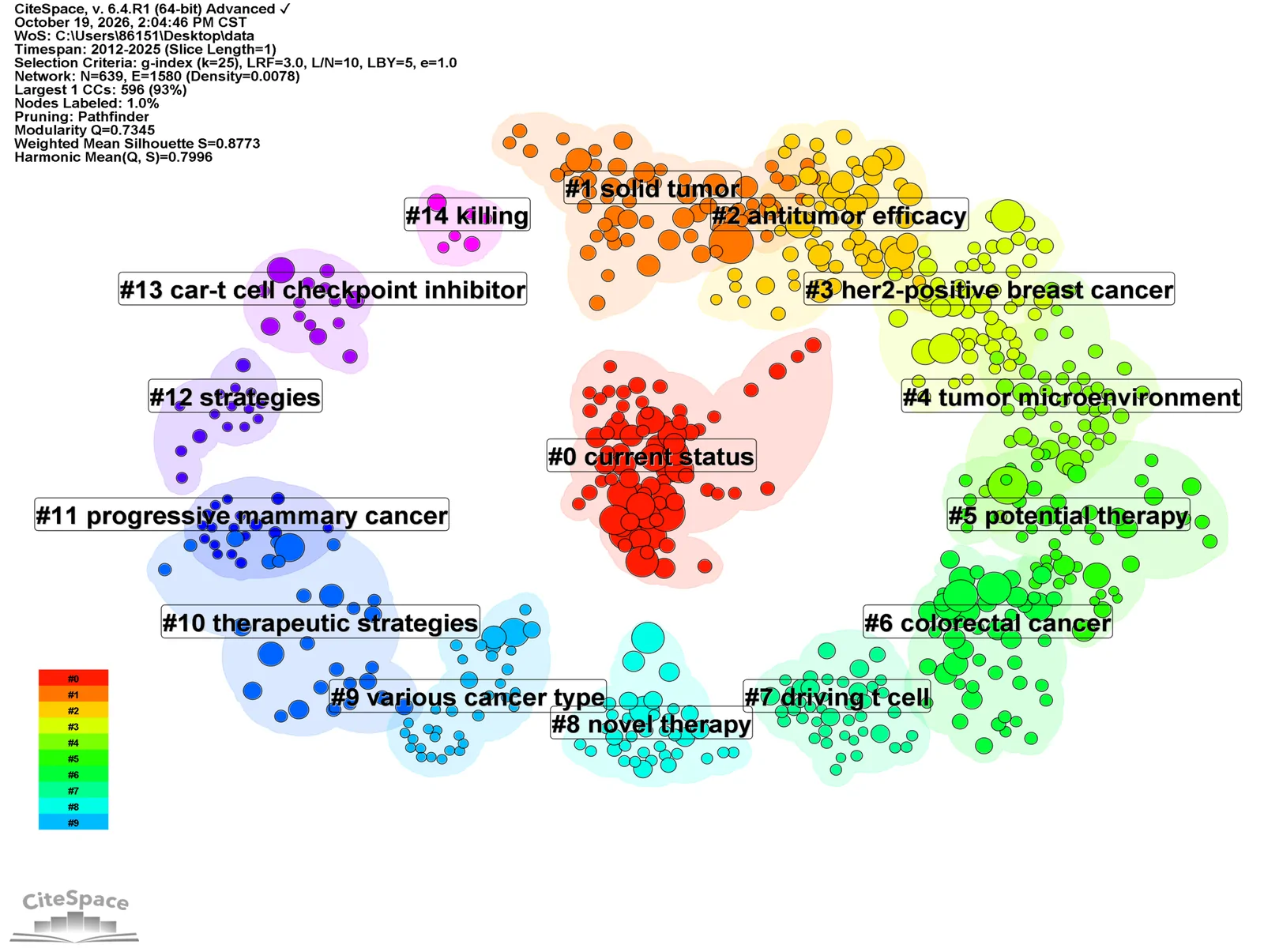
Mapping of the reference co-citation cluster. Co-cited references with high similarity were grouped into the same cluster, and each cluster was labeled by the cited terms extracted from the titles of member references.

## 4. Discussion

Unlike previous narrative reviews that focused on technical details or a single clinical perspective, this study is centered on the results of bibliometrics. It mainly discusses the research trends, collaboration structures, thematic frontiers, and the significance of changes in the field of breast cancer CAR-T therapy.

### 4.1. The evolution and implications of CAR-T technology

The keyword time zone chart shows that in early studies, basic concepts such as “antitumor activity” and “adoptive immunotherapy” were dominant. Subsequently, terms like “chimeric antigen receptor” and “immunotherapy” remained active. In recent years, clinical-related keywords such as “cytokine release syndrome” and “tumor microenvironment” have emerged prominently. The above trend indicates that breast cancer CAR-T research has gradually expanded from the initial stage of designing the CAR structure and verifying its antitumor activity, to directions such as TME, antigen heterogeneity, combined therapy, and clinical toxicity management. The research focus at each stage overlaps, jointly forming the knowledge structure of dynamic evolution in this field.

### 4.2. Research strength and cooperative network

The national and institutional cooperation network reveals that some European institutions play a crucial bridging role within the network. A recent study on CAR-T therapy for solid tumors brought together institutions from the United States, Iran, Russia, and others.^[35]^ It not only covered hematology, immunology, and stem cell research, but also integrated toxicology, pharmacology, virology, and even behavioral science. This transnational and interdisciplinary cooperation model indicates that the clinical translation of CAR-T is no longer merely an immunological issue but a systematic engineering that requires collaboration among multiple countries and disciplines, especially in terms of toxicity management and optimization of clinical translation pathways. The co-citation analysis of the authors and the co-citation analysis of journals further demonstrate that the knowledge base in this field not only comes from the classic research and clinical paradigms of CAR-T in hematological malignancies but also from the research on solid tumor immunotherapy and tumor biology. Although a certain international cooperation network has been formed, there is still room for improvement in substantive cross-institutional and interdisciplinary cooperation, which may be one of the structural factors limiting the rapid transformation of innovative strategies into clinical benefits.

### 4.3. Theme evolution and cutting-edge hotspots

The keyword co-occurrence and clustering analysis identified several research topics, including triple-negative breast cancer, the progression of HER2-positive breast cancer, the TME, drug resistance mechanisms, myeloid-derived suppressor cells, etc. Keywords related to themes such as TME, epithelial–mesenchymal transition, and mesothelin (MSLN) are also the cutting-edge hotspots in this field. MSLN is a potential target for TNBC CAR-T therapy.^[36,37]^ However, MSLN exhibits heterogeneous expression at the protein level and subcellular location, and this heterogeneity is considered the main obstacle hindering the effectiveness of CAR-T therapy. Recently, Wu et al found that by alkalizing the TME, the killing effect of MSLN-CAR-T cells can be effectively enhanced.^[38]^ The research front is gradually shifting towards microenvironment barriers, tumor plasticity, and target antigen selection transfer. These findings provide clues for subsequent mechanism research and the generation of clinical translation hypotheses.

### 4.4. Clinical and translational implications

CAR-T cell therapy has become the standard treatment for patients with relapsed or refractory ALL and several subtypes of B-cell non-Hodgkin’s lymphoma. As early as 2017, Neelapu and colleagues discovered in a clinical phase 2 trial that axicabtagene ciloleucel-CAR T cell therapy could effectively prolong the objective response rate (82%) of patients with refractory large B-cell lymphoma.^[39]^ In 2018, clinical studies showed that the overall response rate of 75 patients with relapsed/refractory B-ALL who received tisagenlecleucel infusion within 3 months could reach 81%.^[40]^ These data indicate that CAR-T has formed a replicable clinical transformation pathway in hematological malignancies. In contrast, the clinical transformation of breast cancer CAR-T is still at an early stage. Tchou et al evaluated the safety and efficacy of mRNA c-Met-CAR-T in metastatic breast cancer in a phase 0 clinical trial (NCT01837602).^[41]^ The results showed that the mRNA c-Met-CAR-T cell injection was well tolerated, and the scheme reached the safety endpoint. However, due to the short expression of CAR in mRNA CAR-T cells, it affected the long-term efficacy of the treatment. Solid tumor CAR-T is generally limited by antigen heterogeneity, iTME, T cell infiltration, and insufficient durability, and the objective response rate is much lower than that of hematological malignancies.^[9,42]^ The above literature data suggest that the clinical transformation of breast cancer CAR-T still requires more early clinical trials for verification, and the visualization analysis of literature metrics will provide research clues for clinical practice.

### 4.5. Limitation

This study still has certain limitations. Firstly, the literature search was limited to the WoSCC, which might have missed relevant studies in other databases such as PubMed, Scopus, and Embase. Secondly, the research was in English, which might introduce language and regional biases. Thirdly, the search strategy was based on titles and abstracts, focusing on “CAR-T” and “breast cancer,” which might have missed synonymous keywords or keywords that appeared only in full texts or non-standardly named studies. Fourthly, in the rapidly evolving field of breast cancer CAR-T, the citation lag effect is particularly prominent. Recent high-impact studies might not be fully reflected in the co-citation analysis. Moreover, the co-citation analysis relies on references and might be affected by citation inflation and self-citations in the reviews. Finally, bibliometrics reflects the publication, keywords, and citation patterns, and cannot directly assess the biological mechanism, clinical efficacy, or safety of breast cancer CAR-T treatment.

### 4.6. Policy and financial implications

From the results of bibliometric analysis, it can be seen that breast cancer CAR-T research is highly dependent on international cooperation, and the research frontiers are concentrated in directions such as TME and epithelial–mesenchymal transition. These directions usually require long-term, high-cost research investment and complex clinical care support. Currently, CAR-T therapy for solid tumors, such as breast cancer, is still in the exploratory research stage. Therefore, continuous and stable support is needed at the policy and financial levels. A cross-institutional and cross-border collaboration network should be established to promote research on target development, safety control, and clinical transformation.

## 5. Conclusion

In conclusion, this study systematically sorted out the development of CAR-T therapy for breast cancer in the past decade using bibliometric methods. The research has expanded from the early stage of CAR structure design to topics such as antigen heterogeneity, iTME, CAR-T cell exhaustion, and targeted therapy. The complexity of these topics indicates that their breakthroughs rely on in-depth collaboration across institutions and disciplines. However, current analysis of the cooperation network shows that substantive collaboration is still insufficient. Future research should focus on optimizing the breast cancer CAR structure, combining targets, and predicting and managing clinical toxicity, in order to promote the clinical translation of CAR-T therapy in solid tumors.

## Author contributions

**Conceptualization:** Xue Liu, Yan Gao, Huabao Xiong.

**Data curation:** Yan Gao, Xue Liu.

**Formal analysis:** Ruiping Jia.

**Funding acquisition:** Xue Liu.

**Investigation:** Zhiqiang Qu, Changying Wang.

**Methodology:** Yan Gao, Huan Liu.

**Software:** Zhiqiang Qu, Yaxin Zhou.

**Visualization:** Ruiping Jia, Huan Liu.

**Writing – original draft:** Xue Liu, Yan Gao.

**Writing – review & editing:** Xue Liu, Huabao Xiong.
